# Cognitive Decline With Low-Dose Procyclidine and Improvement Following Removal of Anticholinergic Burden

**DOI:** 10.1192/bjo.2024.656

**Published:** 2024-08-01

**Authors:** Shaimaa Aboelenien, Vinu Cherian, Joel Philip

**Affiliations:** 1 Essex Partnership University NHS Foundation Trust, Southend-on-Sea, United Kingdom; 2 Essex Partnership University NHS Foundation Trust, Thurrock, United Kingdom

## Abstract

**Aims:**

A recent Cochrane review published in December 2023 concluded that “no trials found that interventions to reduce anticholinergic burden led to any other improvements in cognition compared to usual care”. We describe the case of a 62-year-old lady who developed significant cognitive decline following the initiation of a low dose of procyclidine, which was rapidly reversed upon stopping the medication.

**Methods:**

We present the case of a 62-year-old lady with a diagnosis of schizo-affective disorder, whose symptoms had been stabilized on a regime of lithium carbonate 500mg nocte, sulpiride 400mg BD and fluoxetine 20mg OD. When the patient presented to the outpatient clinic, she was noted to have bilateral coarse tremors and slight cogwheel rigidity. Procyclidine was started at a dose of 5mg OD to manage these extrapyramidal side-effects.

Following this, family members reported that the patient had difficulty initiating and following conversations. Short-term memory was affected and she was observed to have reduced attention span. These problems were reportedly getting worse with time, with a simultaneous decline in functional abilities. She was no longer able to carry out her daily shop, and family members ensured that she was no longer driving as they had concerns about her road safety. She stopped taking procyclidine after 1 month and notably, these problems ceased within one week of stopping the medication.

Cognitive testing confirmed that the patient was cognitively intact after procyclidine was stopped. The patient scored 96 on the ‘Addenbrooke's Cognitive Examination’ scale, which falls within the normal range. The ‘Instrumental Activities of Daily Living’ scale was administered to assess functioning at the time of the cognitive impairment. This returned a score of 1/8, indicating that there was significant functional impairment secondary to cognitive impairment when prescribed procyclidine. The ‘Informant Questionnaire on Cognitive Decline in the Elderly’ was administered to objectively quantify the extent of cognitive decline as noted by family. This returned a score of 4.3, confirming that the patient's cognition had indeed been worse when compared with her baseline.

**Results:**

Our case report highlights the rapid improvement in cognition with the removal of anticholinergic burden in a 62-year-old female. Our report can, therefore, be a harbinger for more robust trials to determine the efficacy of interventions to reduce anticholinergic burden in preserving or improving cognition.

**Conclusion:**

It is important to monitor for any change in cognition when prescribing anticholinergic medication in at-risk individuals.

